# Crimean-Congo Hemorrhagic Fever in Migrant Worker Returning from Oman to India, 2016

**DOI:** 10.3201/eid2306.161950

**Published:** 2017-06

**Authors:** Pragya D. Yadav, Sachin Thacker, Deepak Y. Patil, Rajlaxmi Jain, Devendra T. Mourya

**Affiliations:** National Institute of Virology, Pune, India (P.D. Yadav, D.Y. Patil, R. Jain, D.T. Mourya); JK Hospital,; Bhuj, India (S. Thacker)

**Keywords:** Crimean-Congo hemorrhagic fever, Crimean-Congo hemorrhagic fever virus, imported case, India, Oman, viruses, zoonoses, tick-borne disease, vector-borne infections

## Abstract

In January 2016, a migrant worker who returned home to India after becoming ill in Oman was confirmed to have Crimean-Congo hemorrhagic fever (CCHF). Physicians should include CCHF in the differential diagnosis for patients with hemorrhagic signs and a history of recent travel to any area where CCHF is endemic or prevalent.

Increased international travel has led to the global spread of numerous diseases (*1*). One of these diseases, Crimean-Congo hemorrhagic fever (CCHF), has affected persons in >20 countries in Africa, Asia, southeastern Europe, and the Middle East (*2*). Transmission of CCHF virus (CCHFV) to humans primarily occurs via *Hyalomma* spp. ticks and livestock. Large numbers of nosocomial and sporadic CCHF outbreaks have been reported in humans worldwide, including in India, where information about travel-associated CCHF cases is lacking (*3*). We report on a case of CCHF in a man who returned home to India after becoming ill in Oman.

## The Study

On January 24, 2016, a 33-year-old migrant worker from India became ill while working in Muscat, Oman. He experienced abdominal pain, occasional dysuria, anorexia, nausea, and vomiting. The man, a supervisor on an animal farm, had occasional contact with animals, including cows, goats, horses, and camels. On January 26, he was admitted to a hospital in Muscat and diagnosed with severe thrombocytopenia and acalculous cholecystitis; he was discharged with a referral to the government hospital for further care. On January 27, instead of visiting the government hospital, he traveled to his residence in Gujarat State, India. On January 28, he was admitted to a hospital in Kutch District, Gujarat, with fever; hemorrhagic signs (melena, epistaxis, and hematuria); vomiting; loss of appetite; and altered sensorium. He had a platelet count of 33 × 10^9^/L (reference range 130–400 × 10^9^/L); hemoglobin level of 6.8 g/dL (reference range 14.0–17.4 g/dL); aspartate and alanine aminotransaminase levels of 130 U/L (reference range 0–35 U/L) and 240 U/L (reference range 3–36 U/L), respectively; and prothrombin time of 17.9 sec (reference range 10–13 sec). Results for brain multidetector computed tomography scanning were normal. The patient was administered intravenous ceftriaxone, pantoprazole, somazina citicoline, and cerebroprotein hydrolysate and oral ribavirin.

On illness day 5, the physician sent a clinical sample to the National Institute of Virology in Pune, India, for testing. Results were negative for dengue NS1 antigen and IgM. However, using real-time reverse transcription PCR and IgM ELISA as previously described (*4*), the Institute detected CCHF viral RNA (9.0 × 10^2^ copies/5 μL) and IgM. 

After confirming CCHFV infection in the patient, the hospital placed him in quarantine and implemented strict barrier nursing practices; no secondary cases occurred. Contacts in India were closely monitored for 15 days; all remained asymptomatic. The patient was discharged 13 days after illness onset. A clinical sample tested 15 days after discharge was positive for CCHFV IgG and IgM and negative for viral RNA.

We attempted to isolate CCHFV by inoculating infant CD-1 mice with 20 μL of serum or blood collected from the patient on illness day 5. The mice showed no clinical signs by postinoculation day 7, when we euthanized the mice, harvested brains, made a brain suspension in 1.25% bovine serum albumin in phosphate-buffered saline, and used it to inoculate infant mice. Beginning on postinoculation day 5, these mice began showing neurologic signs, (e.g., hind limb paralysis, circling movement); 1 mouse died. We also attempted to isolate CCHFV by inoculating the brain suppension into Vero CCL-81 cells. We detected high virus loads in the mouse brain suspensions (1.2 × 10^4^ copies/5 μL) and tissue culture fluid (2.3 × 10^4^ copies/5 μL) (*4*).

Using the cell culture–grown virus and previously established protocols (*5*), we sequenced the complete genome (small [S], large [L], and medium [M] segments) of the virus. We used Sequencher 5.4.5 DNA Sequence Analysis Software (Gene Codes Corporation, Ann Arbor, MI, USA) to align sequences and submitted them to GenBank (accession nos. KY213714 [S gene], KY213712 [L gene], KY213713 [M gene]). We used a neighbor-joining algorithm in MEGA6 (http://www.megasoftware.net/) to perform phylogenetic analyses, and we constructed a phylogenetic tree using the sequences of the 3 segments and previously identified representative CCHFV strains.

**Figure 1:**
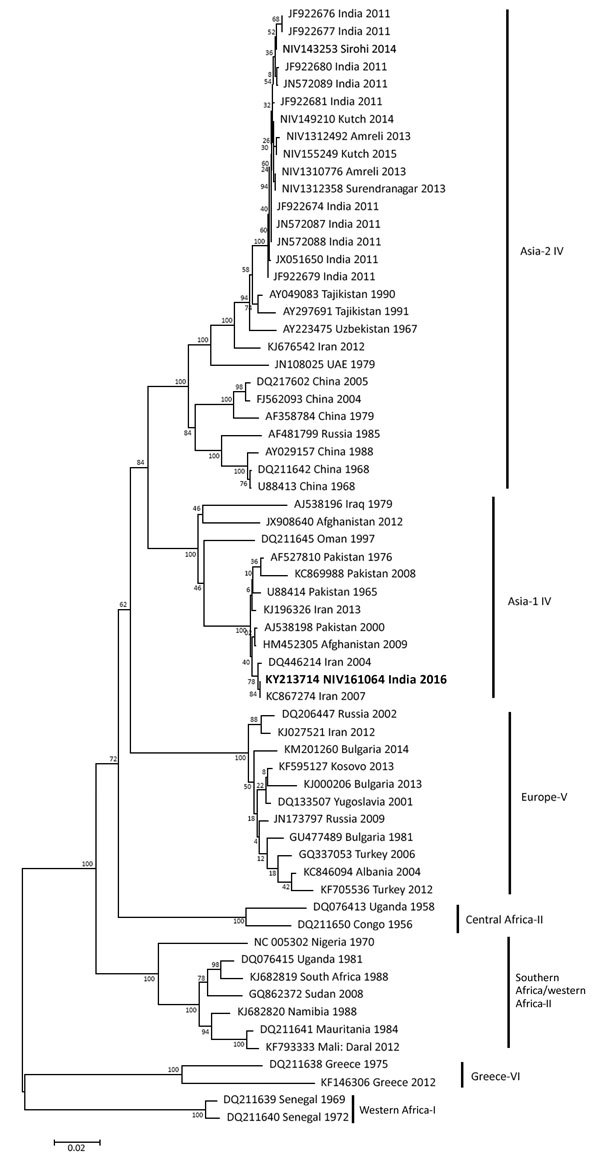
Phylogenetic tree comparing the small gene segment of Crimean-Congo hemorrhagic fever virus (CCHFV) strain isolated in India (bold) with reference CCHFV strains obtained from GenBank. The strain from India, NIV161064, was isolated in 2016 from the serum of a patient who had returned home to India after becoming ill in Oman. Representative reference strains were inferred by the neighbor-joining algorithm in MEGA6 (http://www.megasoftware.net/). Strains are identified by GenBank accession number and location and year of isolation. CCHFV groups are shown on the right. Scale bar indicates number of nucleotide substitutions per site.

**Figure 2:**
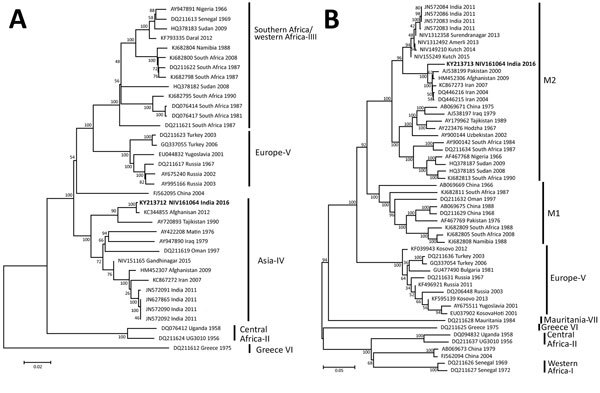
Phylogenetic trees comparing the large (A) and medium (B) gene segments of Crimean-Congo hemorrhagic fever virus (CCHFV) strain isolated in India (bold) with reference CCHFV strains obtained from GenBank. The strain from India, NIV161064, was isolated in 2016 from the serum of a patient who had returned home to India after becoming ill in Oman. Representative reference strains were inferred by the neighbor-joining algorithm in MEGA6 (http://www.megasoftware.net/). Strains are identified by GenBank accession number and location and year of isolation. CCHFV groups are shown on the right. Scale bars indicate number of nucleotide substitutions per site.

By analyzing the S segment, we demonstrated that this CCHFV strain belongs to the Middle East Asia group IV (Asia-1) of CCHFVs, along with strains from Iran, Afghanistan, Pakistan, and Oman (Figure 1); the S segment was closest to those of strains isolated in 2007 in Zehedan, Iran (GenBank accession no. KC867274). The L segment also belongs to the Asia-IV group, along with strains from Afghanistan, Tajikistan, Iraq, and Oman and strains isolated in India in 2011 (Figure 2, panel A); the L segment was closest to that of a strain isolated in 2012 in Afghanistan (GenBank accession no. KC344855). The M segment belongs to type M2 and was closest to the M segment of strains isolated during 2004–2007 in Iran (GenBank accession nos. DQ446216, DQ446215, and KC867273) (Figure 2, panel B).

## Conclusion

During 2011–2016, many CCHF cases were reported from India, especially from Gujarat State, where the virus has been endemic since 2011, and adjoining Rajsthan State, where a few sporadic cases have occurred (*4**,**6**–**11*). Furthermore, we previously conducted a countrywide, cross-sectional serosurvey that showed CCHFV is prevalent among livestock in 22 states and a union territory of India (*9*).

Our findings show that the S segment of the CCHFV isolated in this study shared maximum relatedness with the Middle East Asia group IV isolates, and the M segment belongs to the M2 group, which also includes strains from countries in the Middle East. Thus, the strain isolated from the migrant worker is a combination of the S gene of Asia-I (S–Asia-1) and M2 strains. We also observed parallel clustering of the S and L segments with Asia-IV group viruses. Like the strain in this study, most CCHFVs circulating in the Middle East are a combination of S–Asia-1 and M2 strains (*12*,*13*). However, CCHFV strains reported from different districts of Gujarat during 2011–2015 were a combination of S–Asia-2 and Far East M2 viruses that had different parental origins in the S (from Tajikistan strain TADJ/HU8966) and L and M (from Afghanistan strain Afg09-2990) segments, suggesting that an intragenotypic reassortant sequence entered into India. CCHF cases have been reported from Oman since 1995 (*14*,*15*). However, because sequences have not been reported for recent strains, we could not conduct a more robust phylogenetic analysis. Overall, our phylogenetic analyses substantiate that the case-patient in Gujarat was infected with a CCHFV strain from the Middle East while working in Oman.

The case-patient we report became ill while in Oman and traveled to Gujarat within the incubation period for CCHFV (6–7 days). On illness day 5, the case-patient was hospitalized in Gujarat and confirmed to be infected with CCHFV. Results of serologic testing for IgM corroborate that the patient acquired the infection while in Oman.

Many reports have been made around the world of travelers inadvertently importing diseases from one country to another. Thus, travelers should be made aware of communicable diseases present in countries they visit, and patients should inform doctors if they have a recent travel history. In addition, physicians should consider CCHF in the differential diagnosis of patients who have hemorrhagic signs and have recently returned from any area where CCHFV is endemic or prevalent. Increased international travel will result in further importations of infectious diseases, highlighting the need for worldwide disease surveillance and for implementation of the World Health Organization International Health Regulations (http://www.who.int/topics/international_health_regulations/en/).
